# Psychometric Evaluation of the Computerized Battery for Neuropsychological Evaluation of Children (BENCI) among School Aged Children in the Context of HIV in an Urban Kenyan Setting

**DOI:** 10.1192/j.eurpsy.2023.222

**Published:** 2023-07-19

**Authors:** R. Maina, J. He, A. Abubakar, P.-G. Miguel, M. Kumar, J. Wicherts

**Affiliations:** 1 Methodology and Statistics, Tilburg University, Tilburg, Netherlands; 2 Brain and Mind Institute, Aga Khan University, Kenya, Nairobi; 3 Methodology and Statistics, Tilburg University, Tilburg; 4 Institute of Human Development, Aga Khan University, Kenya, Nairobi, Kenya; 5 Mind, Brain and Behavior Research Center (CIMCYC), University of Granada, Granada, Spain

## Abstract

**Introduction:**

Culturally validated neurocognitive measures for children in Low- and Middle-income Countries are important in the timely and correct identification of neurocognitive impairments. Such measures can inform development of interventions for children exposed to additional vulnerabilities like HIV infection. The Battery for Neuropsychological Evaluationo f Children (BENCI) is an openly available, computerized neuropsychological battery specifically developed to evaluate neurocognitive impairment.

**Objectives:**

This study adapted the BENCI and evaluated its reliability and validity in Kenya.

**Methods:**

The BENCI was adapted using translation and back-translation from Spanish to English language. The psychometric properties were evaluated in a case-control study of 328 children (aged 6 – 14 years) living with HIV and 260 children not living with HIV in Kenya. We assessed reliability, factor structure, and measurement invariance with respect to HIV. Additionally, we examined convergent validity of the BENCI using tests from the Kilifi Toolkit.

**Results:**

Internal consistencies (0.49 < α < 0.97) and test-retest reliabilities (-.34 to .81) were sufficient-to-good for most of the subtests. Convergent validity was supported by significant correlations between the BENCI’s Verbal memory and Kilifi’s Verbal List Learning (r =.41), the BENCI’s Visual memory and Kilifi’s Verbal List Learning (r = .32) and the BENCI’s Planning total time test and Kilifi’s Tower Test (r = -.21) and the BENCI’s Abstract Reasoning test and Kilifi’s Raven’s Progressive Matrix (r = .21). The BENCI subtests highlighted meaningful differences between children living with HIV and those not living with HIV. After some minor adaptions, a confirmatory four-factor model consisting off flexibility, fluency, reasoning and working memory fitted well (χ2 =135.57, DF = 51, N = 604, p < .001, RMSEA = .052, CFI = .944, TLI =.914) and was partially scalar invariant between HIV positive and negative groups.

**Conclusions:**

The English version of the BENCI formally translated for use in Kenya can be further adapted and integrated in clinical and research settings as a valid and reliable cognitive test battery.

**Disclosure of Interest:**

None Declared

